# BMSCs-derived small extracellular vesicles antagonize cerebral endothelial Caveolin-1 driven autophagic degradation of tight-junction proteins to protect blood-brain barrier post-stroke

**DOI:** 10.7150/ijbs.101937

**Published:** 2025-01-01

**Authors:** Yiyang Li, Xingping Quan, Jiacheng Hu, Yan Han, Jinfen Chen, Manfei Zhou, Fan Zhang, Yayue Yang, Mingchun Liao, Bin Wang, Yonghua Zhao

**Affiliations:** 1 Institute of Chinese Medical Sciences, State Key Laboratory of Quality Research in Chinese Medicine, University of Macau, Taipa, Macau SAR, China.; 2 Department of Pharmaceutical Sciences, Faculty of Health Sciences, University of Macau, Taipa, Macau SAR, China.; 3 Guangdong Institute of Intelligence Science and Technology, Zhuhai, Guangdong, China.

**Keywords:** Autophagy, Blood-brain barrier, Caveolin-1, Ischemic stroke, Small extracellular vesicles

## Abstract

Bone marrow mesenchymal stem cells (BMSCs) -derived extracellular vesicles (EVs), especially small EVs (sEVs), were vastly reported to enable multiple restorative effects on ischemic stroke, yet the protective mechanism of blood-brain barrier (BBB) has not been fully illustrated. In the present study, we investigated the therapeutic effects and mechanism of BMSCs-derived sEVs on BBB injury after ischemic stroke. In-vivo, administering sEVs to transient middle cerebral artery occlusion (tMCAo) mice mitigated the brain infarct volume, BBB permeability and neural apoptosis, and improved the cerebral blood flow perfusion and neurological function. Simultaneously, cerebral vascular endothelial overexpressed Caveolin-1 (Cav-1) together with its strong co-localization with autophagosome protein LC3B were suppressed, and ZO-1 and Occludin expressions were enhanced, whose results were consistent with those of oxygen-glucose-deprivation/reperfusion (OGD/R)-insulted brain endothelial cells (BECs) *in vitro*. Furthermore, by employing Cav-1 siRNA and pcDNA3.1 transfection, Co-immunoprecipitation, cycloheximide assay, and molecular docking, it proved that brain endothelial Cav-1 was an essential upstream of autophagy activation, contributing to tight-junction proteins delegation via the autophagy-lysosomal pathway. Altogether, our study demonstrates the novel mechanism of Cav-1-dependent tight-junction proteins autophagic disruption on BBB integrity after ischemic stroke, and BMSC-sEVs treatment can reverse such hazard cascades.

## Introduction

Ischemic stroke has become a secondary death and disability cause worldwide, and over 13 million cases of stroke onset globally, accompanied by sorrowful stress to patients' families and a great financial burden to society 1. However, unsatisfied therapeutic interventions for ischemic stroke remain to be an obstacle for medical measures to advance. Till nowadays, although many novel thrombolysis drugs have emerged for ischemic stroke treatment (e.g. Tenecteplase), recombinant tissue plasminogen activator (r-tPA) treatment within a narrow therapeutic time window (within 4.5 hours from stroke onset) is still the only FDA-approved thrombolysis agent for ischemic stroke patients 2, 3. Moreover, though previous studies revealed that eligible patients identified by advanced imaging can receive intravenous thrombolysis with a longer time window (4.5-9 hours) 4, 5, most of the patients who do not meet the criteria fail to receive due interventions on time, and the potential risk such as intracerebral hemorrhage (ICH) cannot be fully avoided. All the dark sides mentioned above make an extremely low percentage of patients who can benefit from r-tPA administration 1, 6, and the intravenous r-tPA administration strategy has a relatively low recanalization rate (20-50%, dependent on the occluded vessel sites. 7, 8. Another strategy is mechanical thrombectomy within 6 to 24 hours after stroke onset in patients with large vessel occlusion (LVO), however, the criteria for the selection of eligible patients and endovascular risk should also be considered 9. In addition, dual antiplatelet therapy (DAPT), is only suitable for minor stroke patients based on a recent clinical trial 10. Hence, developing novel therapeutics for ischemic stroke is urgent and necessary.

Extracellular vesicles (EVs) secreted by stem cells are widely reported to show robust potential for ischemic stroke treatment, especially those smaller-sized extracellular vesicles (sEVs) which can cross the blood-brain barrier (BBB), maintain BBB integrity, mediate neurovascular remodeling and neuroinflammatory response 11, 12 in central nervous system diseases including ischemic stroke. Compared with their parent stem cells therapy, EVs have the capability to reach similar efficacy with more low immunogenicity, tumor formation concern and ethical issues 13, 14. The possible mechanisms of EVs therapy on ischemic stroke may be attributed to their contained non-coding RNAs and proteome 15, 16, nonetheless, the detailed mechanism of how EVs improve stroke recovery is still ambiguous.

Autophagy is a crucial self-degradation biological process that delicately controls the disruption of proteins and substances via the autophagosome-lysosome pathway, dynamically regulating cells survival and maintaining cellular homeostasis 17. In the acute phase of ischemic stroke, autophagy is a double-edged sword, on the one hand recycling the damaged cellular compartments and toxic substances, while on the other hand aggravating cellular death of neurons, endothelial, and glia 18. In the acute cerebral ischemia phase, autophagy also participates in the maintenance of BBB integrity, which is essential for stroke prognosis. Previous studies have identified that autophagy is responsible for tight junction proteins degradation, inducing BBB disruption 19-21, and notably, a scaffolding protein, Caveolin-1 (Cav-1), was proven to be increased and involved in the sub-cellular redistribution of tight junction proteins 21-25. Apart from the broad bio-regulation process (such as anti-apoptosis, inflammation, and promoting neuro-angiogenesis) of Cav-1 on acute ischemic recovery as widely documented 26, 27, interestingly, recent studies revealed that Cav-1 also directly regulated autophagy in the endothelium, affecting endothelial barrier permeability and function in diverse diseases 28-31, yet, the exact role of Cav-1 affecting BBB integrity by brain endothelial autophagy still awaits to be demonstrated. To better bridge this gap and understand the mechanism of stem cell-derived EVs treatment after ischemic stroke, in this study, we investigated the therapeutic effects of BMSC-derived small EVs (sEVs) on BBB injury in cerebral ischemia/reperfusion model, and firstly illustrated the mechanism was related to Cav-1 mediated autophagic degradation of tight junction protein ZO-1 and Occludin.

## Materials and Methods

### sEVs isolation from bone marrow mesenchymal stem cells (BMSCs)

sEVs were isolated and purified from BMSCs by using ultracentrifugation. Briefly, rat BMSCs (Purchased from Shanghai Zhong Qiao Xin Zhou Biotechnology Co., Ltd) were cultured in 75 cm^2^ flasks with Dulbecco's modified Eagle's medium (DMEM) with 1% penicillin/streptomycin (PS) and 10% fetal bovine serum (FBS) in a density of 2.5 ⅹ10^6^ per flask in an incubator with 37 ℃ and 5% CO_2_. 24 hours later, the culture medium was replaced by EVs-free medium containing EVs-free FBS produced by ultracentrifuged under 100,000 g for 18 h at 4 ℃ as a previous report 32. Cells were allowed for growth for another 72 hours, then, the cells' supernatant was centrifuged under 2000 rpm for 10 minutes and passed through a 0.22 μm filter. Afterward, the supernatant was ultracentrifuged under 110,000 g for 120 mins at 4 ℃ by an ultracentrifuge (XPN-100, Beckman Coulter). The pellet was collected and reconstituted in PBS, and re-ultracentrifuged under 110,000 g for 120 mins at 4 ℃. The final sEVs pellet was collected and diluted in PBS for further experiments. For each time of isolation, 8 flasks of BMSCs (about 2 ⅹ10^7^ in the original seeded cells) were used for isolation and diluted in 1.6 ml PBS. Each 100 μl of the sEVs solution was packed in a 1.5 ml microtube and stored in -80 ℃ till use.

### sEVs nano-size, dosage, phagocytosis, distribution and morphology determination

The size and concentration of sEVs were evaluated by a nanoparticle tracking analysis (NTA) device (Nanosight-NS500, Malvern Panalytical Ltd), and BCA assay by a BCA kit (Biosharp Lifescience, China) was used for sEVs protein concentration evaluation. For sEVs phagocytosis experiment, 5 μM DiI dye (Invitrogen) was incubated with sEVs, and then washed by PBS with ultracentrifugation at 110,000 g for 120 mins. The isolated DiI-labeled sEVs were co-cultured with b. End3 cells under 37 ℃ for 120 mins. The cells were then fixed by 4% paraformaldehyde (PFA), and permeabilized by 0.5% Triton X-100. DAPI (Beyotime Biotechnology, China) was stained to visualize the nucleus, and then samples were observed by a confocal microscope (SP8, Leica). For in-vivo DiI-sEVs distribution, mice were subjected to tMCAo surgery, and 200 μg DiI-sEVs were injected via tail vein, and 6, 12, and 24 hours after injection, mice were perfused, and sacrificed for brain sampling. After slicing into 20 μm frozen slices, the brains were stained with vessel marker Lectin, and neuron marker Tuj-1 before imaging. Furthermore, sEVs morphology was detected by transmission electron microscope (TEM, Hitachi) (Service provided by Servicebio Inc., Wuhan, China).

### OGD/R-insulted brain endothelial cells and neurons and administrations

Mice brain cells line b. End 3 (Purchased from Shanghai Zhong Qiao Xin Zhou Biotechnology Co., Ltd) was cultured with DMEM with 1% PS and 10% FBS, and human neuroblastoma cells line SH-SY5Y were cultured with DMEM/F12 medium with 1% PS and 10% FBS (Gibco). All cells were kept in an incubator with 37 ℃ and 5% CO_2_. For the in-vitro brain endothelial and neuronal oxygen-glucose-deprivation/reperfusion (OGD/R) model, the two kinds of cells medium was replaced by DMEM without glucose (Gibco), and cells cultures were placed under the hypoxia chamber (MIC-101, Billups-Rothenberg Inc., United States) charged with 95% N_2_, and 5% CO_2_ in 37 ℃. 2.5 hours later, the cells were transferred to the normoxia culture condition and replaced the medium with the normal culture medium for 24 hours of re-oxygen. For the sEVs treatment, low (2×10^6^ sEVs/ml, 0.1 μg/ml), medium (2×10^7^ sEVs/ml, 1 μg/ml), and high (2×10^8^ sEVs/ml, 10 μg/ml) dosages of sEVs were added to the cells medium after the OGD/R treatment. For brain endothelial cells autophagy regulation, autophagy inhibitor 3-methyladenine (3-MA, 10 mM, MedChemExpress, United States), and lysosomal inhibitor bafilomycin A1 (bafA1, 100 nM, MedChemExpress, United States) were administered for 1 hour before OGD/R insult, whereas autophagy inducer rapamycin (100 nmol/L, MedChemExpress, United States) was employed 24 hours before OGD/R insult. For cycloheximide (CHX) chase assay, CHX (10 μM, MedChemExpress, United States) was applied in OGD/R insulted b. End 3 cells for 3-12 hours to inhibit proteasome pathway for the degradation of tight junction proteins.

### Transwell assay

To determine the in-vitro brain endothelial barrier permeability, a transwell assay was performed as our previous study 33. After high dosages of sEVs were added to the upper chamber, the transwell cell culture plate was subjected to OGD treatment. Then, medium from upper and lower chambers was collected for detection by a microplate reader (SpectraMax M5) at excitation/ emission: 550 nm/ 572 nm. An equation was provided in Supplemental Method 1. was used for the permeability coefficient determination.

### Autophagic flux measurement

To determine the autophagy flux of brain endothelial cells, pCMV-mCherry-GFP-LC3B (Beyotime Biotechnology, China) was transfected to b. End3 cells seeded in confocal dishes, and cultured for 48h, then after the treatment of sEVs or autophagy regulator, the cells were imaged under a confocal microscope (SP8, Leica).

### Cell viability, live/dead cells staining, and ROS level evaluation

Cell viability was tested by using CCK-8 kit (Beyotime Biotechnology, China), 7000 to 10000 cells were seeded into 96 well plates per well, and after OGD/R and sEVs treatment, 10% CCK-8 solution was added to the wells, and cultured for 2 hours before the plates were read at 450 nm absorbance. Cell viability was determined as the fold of Ctrl groups. Moreover, the live and dead cells rates were evaluated by staining cells with HiPer Calcein AM /PI kit according to the protocol (Mei5 Biotechnology, Co., China), by which live cells were stained green and red for dead cells observed by fluorescent microscope (Lecia, DMi8). The percentage of live cells was calculated. To test the ROS level of cells, the Reactive Oxygen Species Assay Kit (Beyotime Biotechnology, China) with DCFH-DA solution was used, and the labeled ROS green fluorescence was observed and calculated.

### Animal cerebral ischemia/reperfusion modeling and sEVs administration

The transient mice middle cerebral artery occlusion (tMCAo) model was established for ischemic stroke modeling. All animal experiment procedures were conducted following the Animal Research: Reporting of *In Vivo* Experiments (ARRIVE) guidelines and were approved by the Ethics Committee of the University of Macau (Ethics Protocol ID: UMARE-010-2023). Briefly, male C57BL/6 mice (8-10 weeks, 20-25g) were anesthetized by isoflurane (4% for surgical induction, and 1.5-2.5% for maintenance), and an incision was made in the neck of the mouse, and the right common carotid artery (CCA), external carotid artery (ECA), and internal carotid artery (ICA) were exposed. Subsequently, a silicon-coated filament (with a head diameter of 0.19±0.01 mm, Changsha Meyue Bio Co., Ltd, China) was introduced from ECA and advanced to the middle cerebral artery (MCA) to temporarily block the blood flow. Reperfusion was established by withdrawing the filament after 60 minutes. The incision was sutured closed and disinfected. Mice in Sham group was only made the incision without filament insertion. Animals were all kept on a warming surgery pad with a set temperature of 37 ℃ after anesthetization and surgery. sEVs (4×10^9^ particles in 200 μl volume, 200 μg) and the same volume of PBS were intravenously administrated via the tail vein after the reperfusion, respectively. For 14 days of long-term experiments, the same dosage of sEVs was once administrated every 3 days.

### Isolation of brain cortex microvessels

Brain microvessels were isolated as previously reported 34. Briefly, after mice were sacrificed, the brain cortexs were collected and homogenated in MCDB 131 medium (Gibco), and was centrifuged at 2000g under 4°C for 5 mins. The precipitation was resuspended in 15% dextran (MW ~ 70 kDa; Sigma-Aldrich), and centrifugated at 10,000 g for 15 min under 4 °C. Consequently, the red pellets enriched with brain cortex microvessels were obtained.

### Intracerebroventricular (ICV) injection

Small interfering RNA was injected intracerebroventricularly to in-vivo regulate the cerebral expressed level of Cav-1. Briefly, after anesthetized by isoflurane, mice were fixed on a stereotaxic apparatus, and a hole was drilled 1mm right lateral from the midline, and 0.34 mm behind the bregma according to The Mouse Brain in Stereotaxic Coordinates (second edition, Copyright © 200 by ACADEMIC PRESS, United States). Hamilton syringe (Hamilton Co, Reno, NV) with 33G needle was inserted 2.5 mm from the surface of the drilled skull. The prepared 4 μl Cav-1 and negative ctrl siRNA solution was injected at the rate of 0.8 μl/min. After finishing the injection, the needle was kept for 10 minutes and then withdrawn slowly. The incision was closed and disinfected, and mice were placed on the warm pad for recovery. 48 hours later the mice were subjected to tMCAo surgery and further experimental treatments. The Cav-1 and negative ctrl siRNA solution preparation and siRNA sequences were illustrated in the Supplemental Method 2.

### The evaluation of BBB permeability, infarct volume, and cerebral blood flow in tMACo mice

Evans Blue assay was employed to test BBB permeability, and TTC staining was for brain infarction evaluation as previous protocol 33. Briefly, 2% Evans Blue (4 ml/kg) was intravenously injected and allowed to circulate for 1 hour. Then mice were sacrificed by intracardiac PBS perfusion under deep anesthesia. The brains were imaged by IVIS® Spectrum small animal image system (PerkinElmer) at excitation/emission wavelength of 620 nm/710 nm. For Evans blue quantification, brain ischemic hemispheres were homogenized by 50% trichloroacetic acid, and centrifuged at 15,000 g for 15 min under 4 °C. The collected supernatant was mixed with 4 times the volume of ethanol, and the fluorescence intensity was determined by a microplate reader (SpectraMax M5) at the excitation/emission wavelength of 620 nm/710 nm. The Evans Blue standard curve was created to evaluate the Evans Blue concentration. For the assessment of brain infarct volume and edema, brain samples were sliced into 2mm sections and stained by 2% 2,3,5-Triphenyl tetrazolium chloride (TTC, Macklin) for 15 minutes at 37 °C. Brain infarct volume and swelling were calculated by using the equation described in Supplemental Method 3. The cerebral blood flow was determined and analyzed by using a laser speckle PeriCam PSI System (Perimed, Sweden), and the ipsilateral blood flow signal was compared with the contralateral site.

### Neurological function assessment

The modified neurological severity score (mNSS) was employed to evaluate the neurological function deficit of tMCAo mice, and the detailed scoring standard was provided in Supplemental Method 4. For the rotarod test, all mice were trained on the rotarod (Ugo Basile, Italy) three days before the testing, and the speed was set to 10 rpm on day 1, 20 rpm on day 2, and 40 rpm on day 3. All mice were trained for 10 minutes each day. On the testing day, the acceleration program was set from 0 to 45 rpm in 2 minutes. The latency to fall and the rpm at which the mouse fell were recorded. Grip strength was recorded by using a grip strength machine (Bioseb, United States). Mice were placed on a metal grid, and by pulling the tail, the max grip strength was recorded, and the data was corrected by body weight. 3 times of measurements were recorded, and the average was determined for the final result. The mice gait analysis was performed after 14 days of the stroke onset by using the DigiGait™ imaging system (Mouse Specifics, Inc., United States). Mice were trained by running on the treadmill belt (15 cm/s) for 3 days before the experiments, and during the experiment day, the running gait parameters of left forelimb, right forelimb, left hindlimb, and right hindlimb were recorded by image system camera (150-200 frames/s), and analyzed by DigiGait™ Analysis system.

### Western blotting

Protein samples from cells, sEVs and mice brain tissues were collected, and the sub-cellular protein was isolated by ProteoExtract Subcellular Proteome Extraction Kit (539790, Calbiochem), and after protein concentration quantifying by BCA assay, the samples were mixed with 5 X loading buffer, and electrophoretic separated by using the 10-12% SDS-polyacrylamide gel. Afterward, the proteins were transferred to PVDF membrane and blocked by 5% BSA. The primary and secondary antibodies were incubated with the membrane, and the protein band was imaged by using the ChemiDoc™ imaging system (Bio-Rad, United States) after incubated with ECL super sensitive luminescent reagent (Biosharp Lifescience, China). The antibodies used were listed in Supplemental Method 5. The relative protein expression levels were calculated by ImagJ software (ImageJ 1.5, NIH, United States).

### Co-immunoprecipitation

Protein G-Magnetic Beads (MedChemExpress, United States) were conjugated with Anti-Cav-1 antibody or Anti-Normal rabbit IgG antibody for 2 h at 4 ℃, after washed by PBS-T (0.5% Tween-20 in PBS, pH 7.4) for 4 times, the same protein mass of samples (300 μg) in each group was incubated overnight at 4 ℃ with the antibody-conjugated beads. After washing by PBS-T, samples were heated under 95 ℃ for 5 min with loading buffer and separated by electrophoresis as western blotting.

### Immunofluorescence

Mice were sacrificed by intraventricular perfusion by cold PBS slowly under deep anesthesia. The brain samples were isolated and fixed, dehydrated, then sliced into 20 μm frozen slices by a cryostat (CryoStar NX70, Thermo Fisher Scientific, United of States). Cell samples were seeded into confocal dishes and subjected to further experiments. The brain slices and cell samples were fixed by 4% PFA and permeabilized by 0.1% Triton X-100, blocked by 2.5% BSA. Primary and secondary antibodies listed in Supplemental Method 5 were incubated with samples, and then DAPI was stained for the nucleus. All samples were sealed to be imaged by a fluorescent microscope (Lecia, DMi8). The relative fluorescent intensity of the targets was calculated by ImagJ software (ImageJ 1.5, NIH, United States).

### Hematoxylin-Eosin (H&E) staining

Tissues were fixed in 4% PFA overnight and then prepared for paraffin slices. Hematoxylin and Eosin were stained in slices, after washing, the slices were imaged by a light microscope.

### TUNEL, Fluoro-Jade® C (FJC), Nissl and silver glycine staining

Ischemic cerebral and cultured neuronal apoptotic cells were stained by using the One Step TUNEL Apoptosis Assay Kit (Beyotime Biotechnology, China) according to the manufacturer's protocol, and the apoptotic cells were stained in green fluorescence. The relative number of TUNEL^+^ cells was calculated. For degradative neuron staining, the FJC staining kit (Solarbio Science & Technology Co., Ltd, China) was employed according to the manufacturer's protocol, and the degradative neurons were stained in green light. The fluorescent intensity of FJC was calculated. To determine the neuronal morphology after cerebral ischemia, brain paraffin slices were stained by Nissl staining, and silver glycine reagents (Servicebio Inc., China) respectively, and the slices were imaged by a light microscope.

### siRNA and pcDNA3.1 transfection

Caveolin-1 and negative Ctrl siRNA or pcDNA3.1 plasmid were transfected to cells to manipulate the mRNA expression of target gene, and lipofectamine 3000 (Invitrogen, United States) was used for the transfection experiments according to the manufacturer's protocol. Detailed siRNA sequences and pcDNA3.1 plasmid were provided in Supplemental Method 2.

### Immunoelectron microscopy

Brain samples were collected on ice and fixed by 2.5% glutaraldehyde in 4 °C for 24 h, the immunoelectron microscopy was carried out with a TEM imaging system (Hitachi) by (Service provided by Servicebio Inc., China).

### Protein-protein docking

Cav-1 and LC3B interaction was predicted by using an online HDOCK server (http://hdock.phys.hust.edu.cn/) 35. Cav-1 (PDB code: 7SC0) and LC3 (PDB code: 1UGM) crystal structure files were downloaded from Protein Data Bank (PDB, NIH, United States), the top 10 docking models were recognized and listed. The protein docking images were visualized by using PyMOL 2.5.8 software.

### Statistical analysis

All data was processed by GraphPad Prism 8 Software and presented as mean ± SD. Data distribution was assessed by using Shapiro-Wilk normality test. Two-tailed independent Student's t-test was employed for comparisons between two groups, and one-way analysis of variance (ANOVA) followed by the Tukey's post-hoc-test was performed for multiple groups comparison. *P* value of <0.05 was determined statistically significant.

## Results

### BMSC-sEVs isolation and characteristics

**Figure 1:**
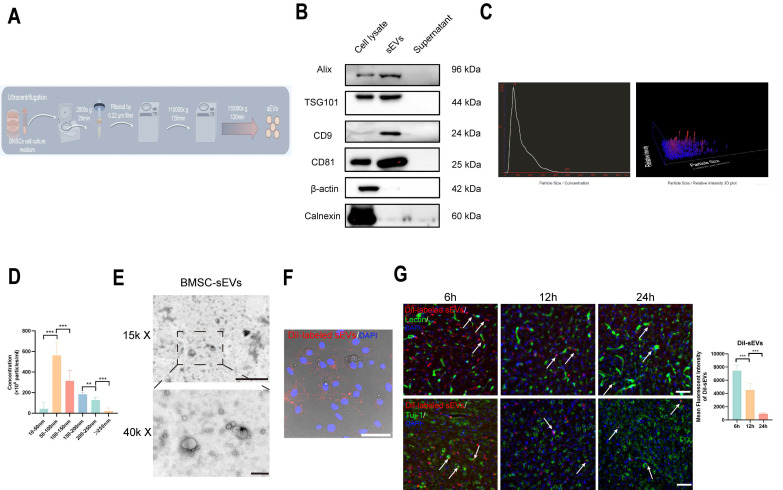
The isolation and identification of BMSC-sEVs. (A) The scheme of sEVs isolation and purification (By Figdraw). (B) Western blotting of sEVs markers in original cells, sEVs, and supernatant after sEVs isolation samples. (C) The NTA analysis of BMSC-sEVs. (D) The size distribution of BMSC-sEVs (n=3). (E) The TEM image of BMSC-sEVs scale bar above: 1 μm, scale bar below: 200 nm. (F) DiI-labeled sEVs phagocytosis by b. End3 cells. Scale bar: 50 μm. (G) DiI-labeled sEVs retention in tMCAo mice brain parenchyma and neurons from 6 to 24h after administration. (Indicated by white arrow) ^***^P < 0.001, ^**^P < 0.01.

BMSC-sEVs were isolated and purified by ultracentrifugation method, the brief experimental procedure was shown in (Fig. 1A), and the sEVs typical marker Alix, TSG101, CD9, and CD81 was highly expressed in BMSC-sEVs, and their parent BMSC cells, and was absent in the supernatant of BMSC culture medium after the sEVs isolation, whereas cell cytoskeletal protein β-actin, and sEVs negative marker Calnexin were mainly expressed in BMSCs (Fig. 1B), and the NTA experiment revealed the isolated sEVs have a high concentrated peak around 50-100 nm (Fig. 1C-D), which was a typical sEVs size range 36. Furthermore, the TEM image of sEVs displayed that the isolated sEVs had a typical cup shape (Fig. 1E), and the DiI-labeled sEVs were phagocytosed by brain endothelial cells (Fig. 1F). Moreover, we administrated DiI-labeled sEVs to tMCAo mice, and the DiI-labeled sEVs could both be reserved in Lectin- labeled brain vessels, and in brain parenchyma beyond vessels from 6 to 24h after sEVs injection, and gradually vanished over time (Fig. 1G). Additionally, DiI-labeled sEVs can retained within 24h in Tuj-1-labeled neurons (Fig. 1G). The above results indicated the isolated sEVs kept a nice phenotype and function.

### BMSC-sEVs treatment alleviated brain infarct volume and neurological function deficit after cerebral ischemia

**Figure 2:**
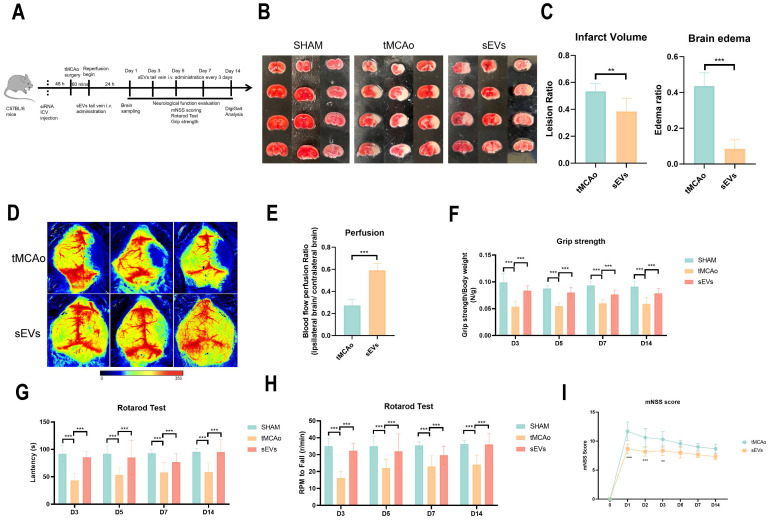
BMSC-sEVs treatment reduced tMCAo insulted mice brain infarct volume and BBB leakage together with neurological function performance. (A) The experimental scheme of animal experiments (B-C) TTC staining and quantification of brain infarct volume and edema of SHAM and tMCAo mice treated with sEVs (n=6). (D-E) Blood flow perfusion of tMCAo mice treated with sEVs by laser speckle (n=6). (F) Grip strength tests of SHAM and tMCAo mice treated with sEVs from 3 to 14 days after surgery (n=6-12). (G-H) Rotarod tests of SHAM and tMCAo mice treated with sEVs from 3 to 14 days after surgery (n=6). (I) mNSS scoring of tMCAo mice treated with sEVs from 1 to 14 days after surgery (n=6-13). ^***^P < 0.001, ^**^P < 0.01.

**Figure 3:**
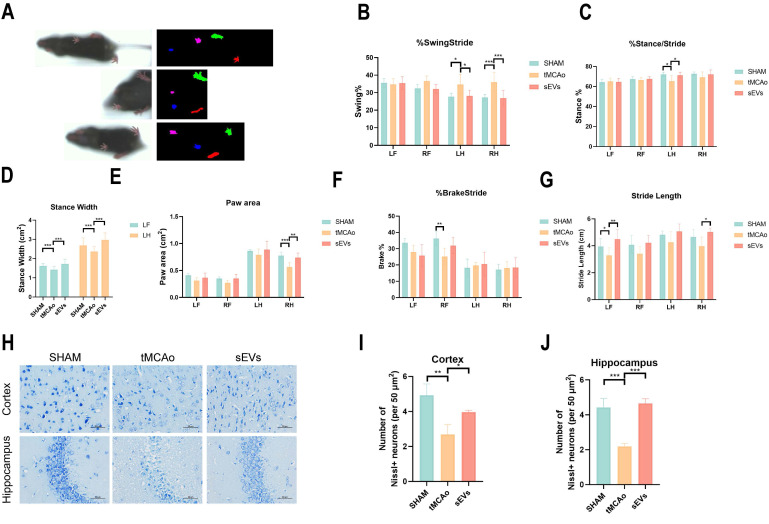
BMSC-sEVs treatment improved the long-term neurological function of tMCAo mice. (A) DigiGait recorded paw print characters of mice in different groups. (B-G) Gait parameters analyzed by the DigiGait system on the 14-day post-surgery. LF: Left forelimb, RF: Right forelimb, LH: Left hindlimb, RH: Right hindlimb. (H-J) Nissl staining and quantification in brain cortex and hippocampus of tMCAo mice treated with sEVs (n=3). Scale bar: 50 μm. ^***^P < 0.001, ^**^P < 0.01, ^*^P < 0.05.

To illustrate BMSC-sEVs therapeutic efficacies, we administrated BMSC-sEVs to tMCAo mice for 14 days treatment after cerebral ischemia onset (Fig. 2A). The results indicated sEVs treatment significantly decreased the brain infarct volume and swelling after 24 hours tMCAo (Fig. 2B-C). Meanwhile, the cerebral blood flow determined by laser speckle imaging was highly recovered in sEVs-treated tMCAo mice (Fig. 2D-E). Inspiringly, in the evaluation of the long-term neurological function in tMCAo mice, BMSC-sEVs treatment obviously enhanced the grip strength, and the performance in the rotarod test, together with the better mNSS scoring during 14 days of cerebral ischemia (Fig. 2F-I). To systematically assess the motor nerve function of tMCAo mice by sEVs treatment, we utilized the DigiGait™ imaging system to analyze the gait abnormality of mice at day 14. The tMCAo mice displayed abnormal gait when compared with the sham group, and sEVs treatment recovered the overall mice gait performance (Fig. 3A). In detail, the abnormality of the left hind limb and right hind limb swing percentage, left hind limb stance percentage, left fore and hind limb stance width, right hind limb paw area, and left fore and right hind limb stride length in tMCAo mice were significantly recovered in sEVs group, however, there is no significant recovery effect by sEVs treatment in brake percentage parament in all limbs (Fig. 3B-G). We further applied nissl staining in mice brain slices to characterize neurons in the cortex and hippocampus, and the number of nissl body-positive neurons was deficient, whereas partly restored in the sEVs treatment group (Fig. 3H-J). Similarly, the silver glycine staining results showed consistency with those of nissl staining (Supplemental Fig. 1). Additionally, we also utilized H&E staining for the hearts, livers, spleens, lungs, and kidneys after sEVs administration in tMCAo mice after 14 days, and it showed there was no obvious occurrence of pathological damages (Supplemental Fig. 2).

### BMSC-sEVs treatment attenuated neuronal injury

**Figure 4:**
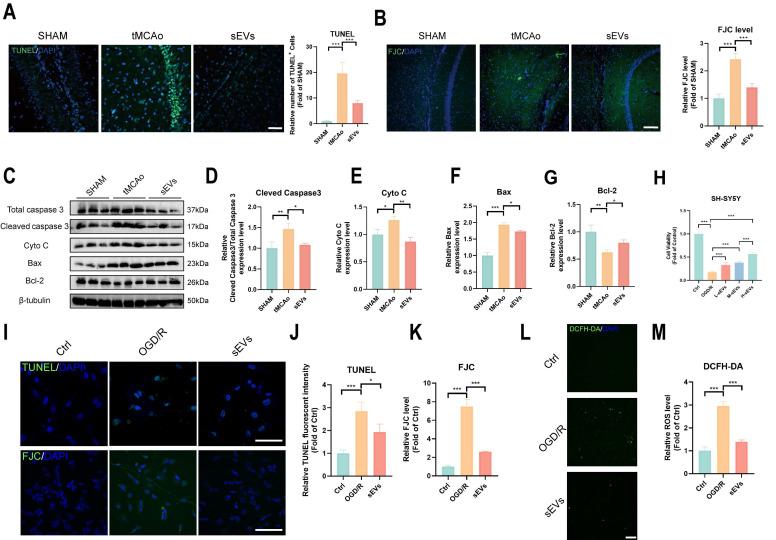
BMSC-sEVs treatment attenuated neuronal apoptosis after cerebral ischemia. (A) TUNEL staining and quantification of mice brain slices in different groups (n=3). Scale bar: 50 μm. (B) FJC staining and quantification of mice brain slices in different groups (n=3) Scale bar: 100 μm. (C-G) Western blotting of Caspase3, CytoC, Bax and Bcl-2 expression in tMCAo mice brain samples and quantification (n=3). (H) Cell viability of SH-SHY5Y cells after sEVs treatments by CCK-8 assay (n=6). (I-K) TUNEL and FJC staining and quantification of SH-SY5Y cells after OGD/R and sEVs treatment (n=4) Scale bar: 100 μm. (L-M) DCFH-DA labeled ROS activity and quantification in SH-SY5Y cells after OGD/R and sEVs treatment (n=4). Scale bar: 200 μm. ^***^P < 0.001, ^**^P < 0.01, ^*^P < 0.05.

Furthermore, we investigated the neural protection by BMSC-sEVs treatment. The increased TUNEL-positive apoptotic cells and FJC-labeled degradative neurons were both inhibited by sEVs treatment in tMCAo mice (Fig. 4A-B). The pro-apoptosis protein cleaved caspase 3, Bax, and cytochrome c were downregulated, and the anti-apoptosis protein Bcl-2 was upregulated after sEVs treatment (Fig. 4C-G). Moreover, we treated OGD/R insulted neuronal cell lines SH-SY5Y with low, medium, and high dosages of sEVs, and figured out that the sEVs treatment significantly recovered the neuron viability in a dose-dependent manner (Fig. 4H). The high dosage of sEVs treatment obviously decreased the number of apoptotic neurons marked by TUNEL staining and the neuron degeneration by FJC staining in OGD/R-insulted neurons (Fig.4I-K). At the same time, the high ROS production was also suppressed by sEVs therapeutic (Fig. 4L-M).

### BMSC-sEVs treatment decreased BBB permeability and upregulated tight-junction proteins expressions

**Figure 5:**
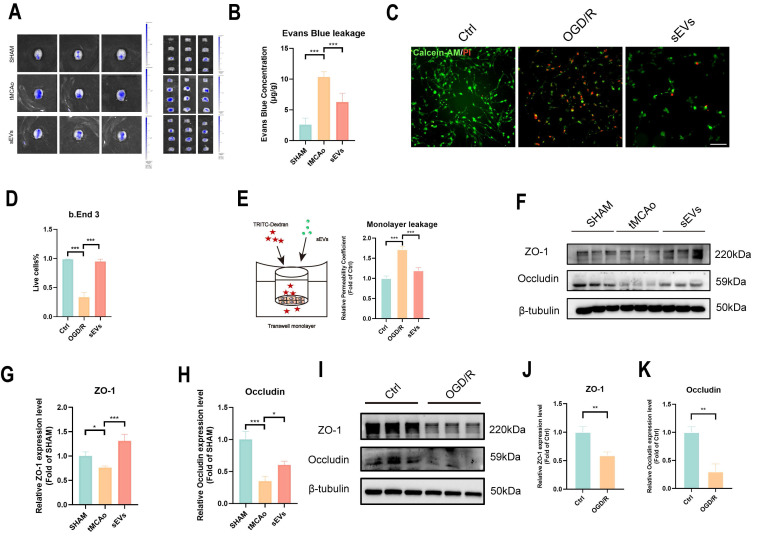
BMSC-sEVs treatment harnessed BBB leakage and recovered tight junction proteins in tMCAo mice. (A-B) Evans blue leakage of SHAM and tMCAo mice treated with sEVs (n=6). (C-D) Live/dead cell staining of b. End3 cells and quantification (n=5) Scale bar: 50 μm. (E) Transwell assay of brain endothelial cells monolayer permeability analysis (n=4). (F-H) Western blotting of ZO-1 and Occludin expressions in tMCAo mice brain microvessel and quantification (n=3). (I-K) Western blotting of ZO-1 and Occludin expression in b. End3 cells after OGD/R and quantification (n=3). ^***^P < 0.001, ^**^P < 0.01, ^*^P < 0.05.

**Figure 6:**
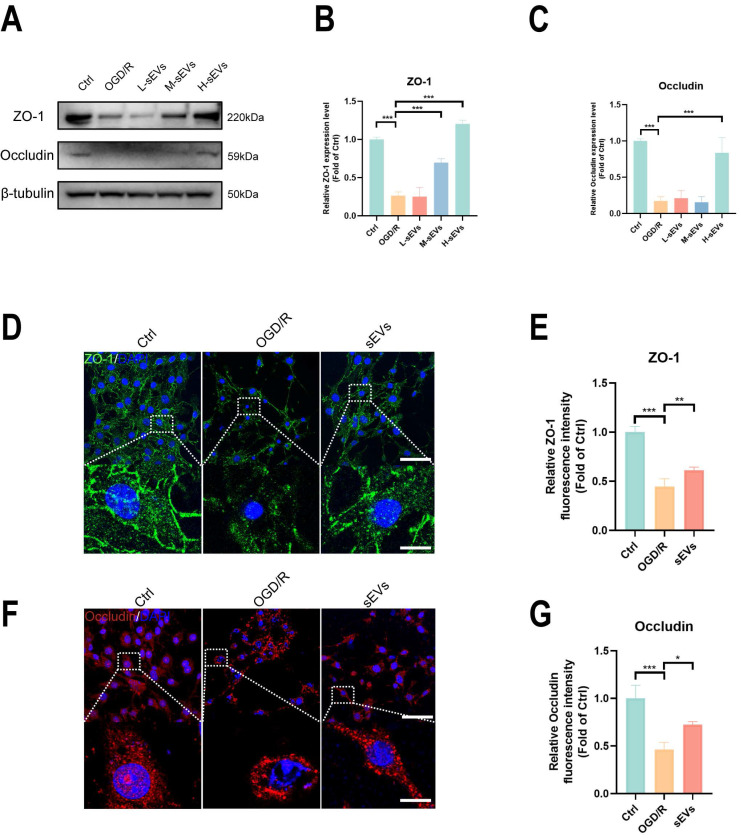
BMSC-sEVs treatment improved tight junction proteins expression in OGD/R insulted b. End3 cells. (A-C) Western blotting of ZO-1 and Occludin expression in b. End3 cells after OGD/R and sEVs treatment and quantification (n=3). (D-G) Immunofluorescence and quantification of ZO-1 and Occludin in brain endothelial cells after OGD/R and sEVs treatment (n=4) Scale bar: 50 μm; 2.5 μm in zoomed image. ^***^P < 0.001, ^**^P < 0.01, ^*^P < 0.05.

In tMCAo mice, the drastic rise of Evans Blue across-BBB leakage was hindered after sEVs treatment (Fig. 5A-B). In addition, we imaged and examined the viability of brain endothelial cells subjected to OGD/R, and the result suggested that the treatment of sEVs enhanced the live cells percentage (Fig. 5C-D). The barrier permeability of the brain endothelial cells monolayer after OGD/R was evaluated by using transwell assay, and the sEVs treatment significantly restrained the upregulated monolayer barrier leakage (Fig.5E). In addition, we evaluated the tight junction protein ZO-1 and Occudin expression in brain microvessels isolated from tMCAo mice, and it showed the expressions of ZO-1 and Occludin were both downregulated, which were reversed in sEVs administrated group 24 hours after modeling (Fig 5F-H). *In vitro*, it suggested that tight junction proteins ZO-1 and Occludin expressions were decreased after OGD/R insult (Fig. 5I-K). and the treatment of sEVs also uplifted the expression levels of these two tight junction proteins, dose-dependently in OGD/R insulted brain endothelial cells (Fig. 6A-C). The immunofluorescent staining was in compliance with the western blotting results (Fig. 6D-G).

### BMSC-sEVs treatment suppressed the autophagy of brain endothelial cells against the degradation of ZO-1 and Occludin

**Figure 7:**
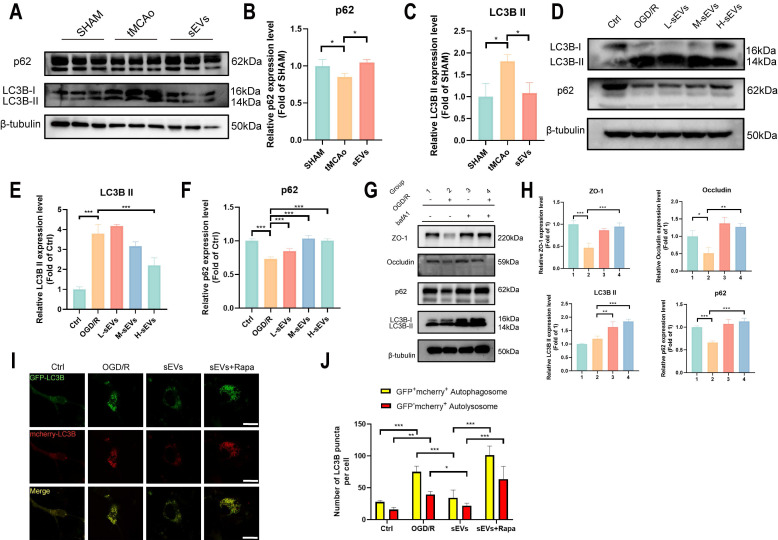
BMSC-sEVs inhibited brain endothelial cells' autophagy flux in-vitro. (A-C) Western blotting of LC3B, and p62 expressions in tMCAo mice brain microvessel and quantification (n=3). (D-F) Western blotting of LC3B and p62 expression in b. End3 cells after OGD/R and quantification (n=3). (G-H) Western blotting of LC3B, p62, and ZO-1/Occludin expression in b. End3 cells after OGD/R co-treated with bafA1 and quantification (n=3). (I-J) Autophagy flux visualized by GFP-RFP-LC3B transfection in b. End3 cells after OGD/R treated with sEVs and rapamycin (n=6) Scale bar: 20 μm. ^***^P < 0.001, ^**^P < 0.01, ^*^P < 0.05.

Autophagy is an important pathway for protein clearance and has been proven to damage tight junctions after cerebral ischemia 20, 37. To illustrate the influence of brain endothelial cells autophagy on BBB integrity and verify whether sEVs therapeutic regulated this pathological abnormality, we examined the autophagosome protein LC3B and autophagic degradation substrates p62 *in vivo* and *in vitro*. In tMCAo modeled mice brain microvessels. LC3B and p62 expressions indicated the activation of microvascular autophagy, and all these trends were reversed in sEVs administrated group after 24 hours modeling (Fig 7A-C). *In vitro*, the western blotting results revealed that LC3B II expression was upregulated after OGD/R, and in contrast, p62 was downregulated in brain endothelial cells, and interestingly, these trends of enhanced autophagy can be reversed by sEVs treatment (Fig. 7D-F). The high dosage of sEVs significantly reduced the LC3B II expression, whereas low, medium and high dosage sEVs all intensified the p62 expression (Fig. 7D-F).

**Figure 8:**
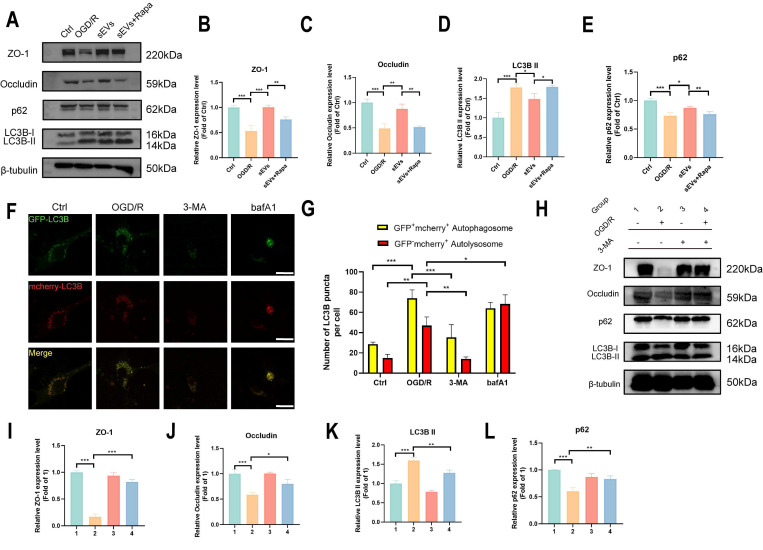
Autophagy is essential for ZO-1 and Occludin degradation after cerebral ischemia. (A-E) Western blotting of ZO-1/Occludin, LC3B and p62 expression in b. End3 cells treated with sEVs and rapamycin after OGD/R and quantification (n=3). (F-G) Autophagy flux visualized by GFP-RFP-LC3B transfection in b. End3 cells after OGD/R treated with 3-MA and bafA1 (n=6) Scale bar: 20 μm. (H-L) Western blotting of ZO-1/Occludin, LC3B and p62 expression in b. End3 cells treated with 3-MA after OGD/R and quantification (n=3). ^***^P < 0.001, ^**^P < 0.01, ^*^P < 0.05.

Subsequently, we investigated if the degradation of ZO-1 and Occludin was autophagy-lysosomal pathway dependent. We applied an autolysosome blocker bafA1, and tested the ZO-1 and Occludin, together with autophagy indicator LC3B and p62 expressions after OGD/R in brain endothelial cells. A restorative trend of ZO-1 and Occludin was observed in bafA1 treating group after OGD/R, together with an upregulation of LC3B II and p62, indicating inhibiting autolysosome pathway rescued the tight junction proteins degradation after OGD/R injury (Fig. 7G-H). Furthermore, the enhanced autophagy flux after OGD/R observed by using GFP-mcherry-LC3B plasmid transfection was remarkably reversed by sEVs treatment, whereas the therapeutic effect was abolished by autophagy enhancer rapamycin administration (Fig. 7I-J). Similarly, the immunofluorescence of lysosome marker LAMP-1 was observed after OGD/R and reduced by sEVs treatment (Supplemental Fig. 3). The western blotting results also indicated the enhanced efficacy of sEVs treatment on OGD/R insulted endothelial tight junction proteins expression was compromised after the cells were administered rapamycin, additionally, and the rise of LC3B II and the decline of p62 simultaneously occurred (Fig. 8A-E). To extensively illustrate the role of early-phase autophagy on ZO-1 and Occludin, we utilized an early-phase autophagy inhibitor 3-MA and lysosome inhibitor bafA1 to OGD/R induced brain endothelial cells, and the autophagy flux test displayed a sharp decrease of both red and yellow puncta number after 3-MA intervention, whereas an upregulation of both fluorescence puncta was observed after bafA1 treatment (Fig. 8F-G). The results indicated the successful manipulation of autophagy by two different autophagy inhibitors. Interestingly, the 3-MA treatment inhibited the LC3B II expression, restored the p62 expression, and at the same time significantly enhanced the ZO-1 and Occludin expressions in OGD/R insulted brain endothelial cells (Fig. 8H-L). The autophagy inhibitors demonstrated similar therapeutic effects on tight junction proteins and autophagy compared to sEVs treatment. Collectively, the results demonstrated the sEVs treatment intervened with the autophagy-lysosome dependent ZO-1 and Occludin degradation.

### BMSC-sEVs treatment regulated Cav-1-dependent autophagic degradation of ZO-1 and Occludin

**Figure 9:**
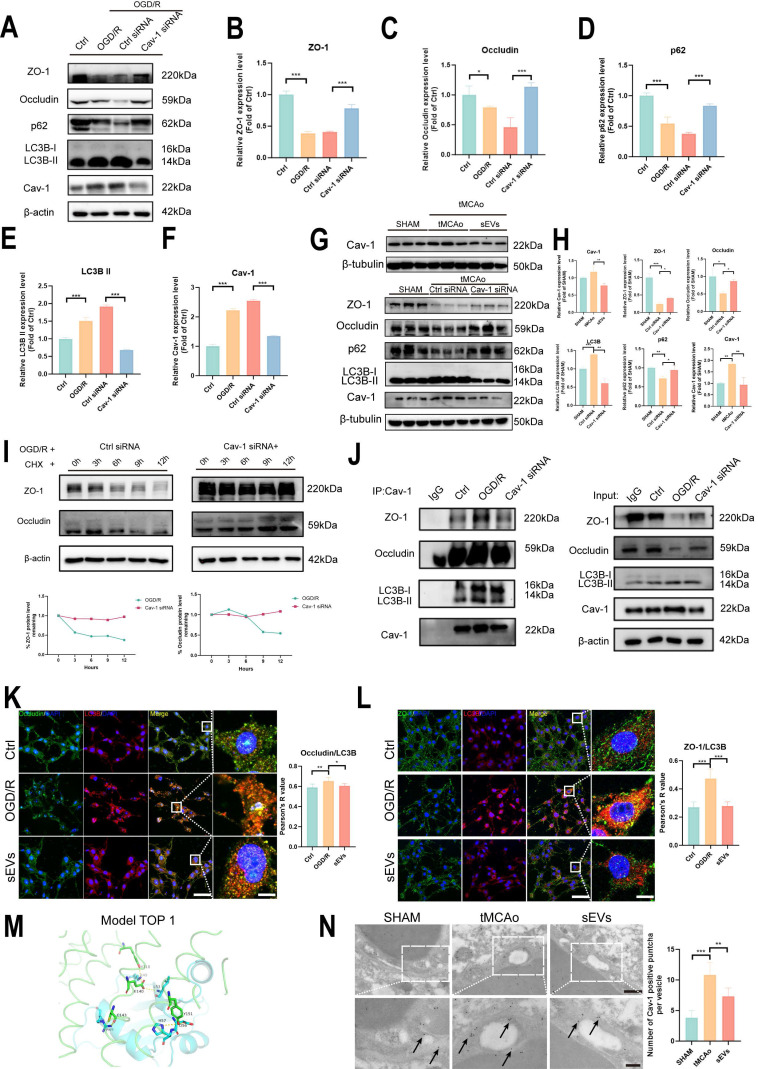
BMSC-sEVs treatment regulated Cav-1 mediated ZO-1 and Occludin autophagic degradation after cerebral ischemia. (A-F) Western blotting and quantification of ZO-1/Occludin, LC3B, p62, and Cav-1 expression in b. End3 cells after OGD/R and Cav-1 siRNA transfection (n=3). (G-H) Western blotting of Cav-1 and ZO-1/Occludin, LC3B, and p62 expression in brain microvessels of tMCAo mice treated by sEVs or ICV injected Cav-1 siRNA (n=3). (I) CHX assay for ZO-1 and Occludin degradation after Cav-1 siRNA transfection in OGD/R insulted b. End3 cells. (J) Co-ip for verifying the interaction between Cav-1 and ZO-1/Occludin or LC3B. (K) Immunofluorescence and Pearson's R co-localization analysis of Occludin and LC3B in b. End3 cells after OGD/R and sEVs treatment (n=4). Scale bar: 50 μm, and 25 μm in zoomed images. (L) Immunofluorescence and Pearson's R co-localization analysis of ZO-1 and LC3B in b. End3 cells after OGD/R and sEVs treatment (n=4). Scale bar: 50 μm, and 25 μm in zoomed images. (M) Protein-protein docking top 1 model of LC3B (Blue) and Cav-1 (Green). (N) Immunoelectron microscopy image of Cav-1 in mice brain microvessels parts, and the quantification of the Cac-1 positive puncta (black arrow indicated) (n=6) Scale bar: 500 nm and 200 nm in zoomed images. ^***^P < 0.001, ^**^P < 0.01, ^*^P < 0.05.

Lastly, we questioned in which pathway BMSC-sEVs mediated autophagy-dependent ZO-1 and Occludin degradation and focused on Cav-1 and its relationship with autophagy. We employed Cav-1 siRNA to knock down the Cav-1 expression in-vitro. The effectiveness of our designed Cav-1 siRNA sequences was presented as the Cav-1 significant knockdown by western blotting, and the siRNA FAM label was positive in brain endothelial after 48 h after transfection (Supplemental Fig. 4). In accordance with the knockdown of Cav-1 in OGD/R-subjected brain endothelial cells, the ZO-1 and Occludin were both recovered, and significant inhibition of autophagy was observed. The result indicated that Cav-1 itself regulated the autophagy process and affected the expression of tight junction proteins (Fig. 9A-F). Subsequently, in-vivo experiment, the increased Cav-1 expression was verified in tMCAo mice brain microvessels and downregulated by sEVs treatment (Fig. 9G-H), similarly, in-vitro OGD induced expression upregulation of Cav-1 in b. End3 cells were decreased dose-dependently after sEVs treatment, especially by the high dosage sEVs (Supplemental Fig. 5). Furthermore, we injected the cholesterol-modified Cav-1 siRNA by ICV approach and examined the tight junction and autophagy proteins expression in ischemic mice brain microvessels after 24 h cerebral ischemia. The increased Cav-1 expression showed a decline in the Cav-1 siRNA administrated tMCAo mice group, when compared with the Ctrl siRNA group, accompanied by a significant restoration of ZO-1 and Occludin, and inhibition of autophagy (Fig. 9G-H). To confirm the degradation of ZO-1 and Occludin was by Cav-1 dependent manner, we applied cycloheximide (CHX) chase assay, and the knockdown of Cav-1 in OGD/R insulted brain endothelial cells. The expressions of ZO-1 and Occludin were gradually decreased over time after CHX treatment, whereas Cav-1 siRNA transfection abolished the declining trend of these two tight junction proteins expression (Fig. 9I). The result indicated that Cav-1 was responsible for ZO-1 and Occludin degradation via the non-proteasomal pathway. Consequently, in order to verify whether sEVs treatment intervened the crosstalk among Cav-1, LC3B, and tight junction proteins, Co-ip experiment validated that the interaction between Cav-1 with ZO-1/Occludin and LC3B was enhanced after OGD/R, whereas downregulated by Cav-1 knockdown (Fig. 9J), and the immunofluorescence results indicated the co-localization between ZO-1/Occludin and LC3B was dramatically upregulated after OGD/R in brain endothelial cells, whereas partly abolished by sEVs treatment (Fig. 9K-L). As we proved the strong autophagy regulation by Cav-1, we next demonstrated the crosstalk between Cav-1 and LC3B. The subcellular protein distribution experiment presented that, after OGD/R, the co-distributions of ZO-1/Occludin, Cav-1, and LC3B were obviously decreased in cytoskeleton fraction, however, accumulated in membrane and cytoplasm fractions, interestingly, this abnormal trend was reversed by sEVs treatment in brain endothelial cells (Supplemental Fig. 6). The in-vitro co-localization of Cav-1 and LC3B was also observed after OGD/R and rescued by sEVs (Supplemental Fig. 7). Next, the binding of Cav-1 and LC3B was predicted by using HDOCK, and the Top 1 binding model was presented (Fig. 9M). All the Top 10 binding models, the docking confidence, and scores were all listed in (Supplemental Fig. 8). In addition, we overexpressed Cav-1 in b.End3 cells after OGD/R and sEVs treatment. The Cav-1 artificial upregulation partly abolished the sEVs' recovery on ZO-1 and Occludin (Supplemental Fig. 9). Furthermore, the number of Cav-1 labeled puncta located in brain endothelial Caveolae vesicles imaged by immunoelectron microscopy was significantly upregulated in tMCAo mice, and the density of the puncta was relatively lower in sEVs administrated mice group (Fig. 9N). All our results suggested that BMSC-sEVs could regulate Cav-1-mediated autophagic degradation of ZO-1 and Occludin after cerebral ischemia.

## Discussion

In the current study, we utilized in-vitro OGD insulted brain endothelial cells and neurons together with an in-vivo tMCAo mice model to demonstrate that BMSC-sEVs treatment effectively restored tight junction protein ZO-1 and Occludin expression levels together with protecting the injured neurons after cerebral ischemia, which contributed to the long-term neurological function amelioration in tMCAo mice. By employing different phase autophagy regulators and Cav-1 siRNA and pcDNA3.1 plasmid, we verified that the therapeutic efficacy of BMSC-sEVs on BBB integrity was Caveolin-1-dependent autophagic degradation of ZO-1 and Occludin after ischemic stroke.

Autophagy is a comprehensive biological cell self-eating progress, and it has multiple roles after ischemic stroke. After cerebral ischemia, autophagy can be an adaptive process for deficient organelles and protein clearance, thus maintaining homeostasis, and facilitating recovery, while on the other hand, uncontrolled excessive autophagy contributes to no benefit but, the activation of endoplasmic reticulum (ER) stress, accumulation of ROS, and mitochondrial deficit can lag restoration and damage of cerebral cells 38, 39. Not only in neurons, but autophagy in brain endothelial cells is also important for their viability, especially affecting BBB permeability 38. The over-activated autophagy degrades tight junction proteins, directly intensifies the BBB permeability after ischemic stroke, and worsens the prognosis 19. Developing effective therapeutic interventions to regulate the over-activated autophagy after ischemic stroke can be a novel and promising way for ischemic stroke treatment.

sEVs have been extensively documented to treat ischemic stroke via regulating excessive neuronal autophagy after ischemic stroke. For instance, human-induced pluripotent stem cells derived sEVs inhibited autophagy via STAT3 activation in MCAo-induced rats, improving rats' cerebral angiogenesis and long-term neurological function after cerebral ischemia 40. Similarly, adipose-derived stem cells inhibited neuronal autophagy in cerebral ischemia mice via miR-25 dependent pathway, which exerted neuroprotection 41. For brain endothelial cells' autophagy regulation, healthy rat serum-originated sEVs downregulated brain endothelial autophagy in MCAo rat, and OGD modeled b. End3 cells, further mediated BBB tight junction protection after ischemic stroke 42. Apart from autophagy regulation, sEVs treatment can directly compose BBB protection, apoptosis inhibition and inflammation suppressing, and promoting angio-neurogenesis after ischemic stroke 15, 43. Compared with traditional stem cell transplantation stroke therapeutics and neuroactive agents, sEVs can cross BBB, and showed low immunogenicity and a relatively low risk of thrombosis 43, 44 which provided a novel solution for stroke treatment. However, the detailed mechanism of how sEVs affect tight junction disruption via autophagy is unclear.

Physiologically, tight junction proteins remain in the plasma membrane to maintain endothelial barrier integrity, and under stimulus, the internalized tight junction proteins were subjected to complex endosomal sorting pathways for recycling or degradation 45, Caveolae-mediated internalization of tight junction proteins were previously reported as one of the predominant endocytosis pathway in brain endothelial cells 25, 45, 46, and the Cav-1 phosphorylation mediated the generation of caveolae, and driven the endocytosis 47, 48. The endocytosed tight junction proteins were then subjected to caveosomes or endosome-dependent sorting and degraded by lysosome or recycled to the membrane 45, 49. Cav-1 was previously reported to translocate ZO-1 and Occludin, intensify the BBB permeability 20, 21, 25, and our previous study also illustrated Cav-1 regulated ZO-1 and Claudin-5 by endocytosis after ischemic stroke 23. The degradation pathways of tight junction proteins are mainly divided into two parts, one is ubiquitin-dependent pathway, after tight junction proteins ubiquitination, they can be degraded by proteasome or lysosome, and Claudin-5 was reported to be degraded via ubiquitin-proteasome routine 45, 50. Another important routine is the autophagy-lysosome pathway, the autophagosome wraps endocytosed tight junction proteins, and fuses with the lysosome to accomplish the degradation, for example, Claudin-2, Occludin and ZO-1 were reported to be degraded via autophagy in intestinal epithelial barrier 51-53. And in BBB damage after ischemic stroke, ZO-1 and Claudin-5 were both documented to be degraded via autophagy-lysosome 21, 22, which were in accord with our finding to find ZO-1 and Occludin were damaged via autophagy-lysosome pathway after ischemic stroke.

On the other hand, Cav-1 involves multiple pathways related to BBB permeability, neuron survival, apoptosis, and autophagy after ischemic stroke 26, 54, 55, and we also revealed that the Cav-1/CD147/VEGFR2/MMP pathway was responsible for BBB disruption and rat neurological function deficit after cerebral ischemia 33. The phosphorylation of Cav-1 bound with BECN1 and thus activated autophagy after cerebral ischemia 56. However, the effect of Cav-1 on autophagy is controversial, in other non-cerebral diseases, Cav-1 was also reported to inhibit autophagy 29, 30, 57. Based on previous work, we endeavored to explore whether Cav-1 not only internalized ZO-1 and Occludin on membrane of brain endothelial cells, but also affected their downstream autolysosome degradation.

Our results firstly revealed that BMSC-derived sEVs inhibited the degradation of tight junction protein ZO-1 and Occludin in brain endothelial cells through the autophagy-lysosomal pathway. We applied 3-MA, a PI3K inhibitor to inhibit early-phased autophagy, and bafA1, a lysosome inhibitor to suppress late-phased autolysosome. The two kinds of inhibitors protected ZO-1 and Occludin in OGD/R insulted brain endothelial cells with the two different autophagy inhibition characters. The Cav-1 knockdown applied *in vitro* and *in vivo* significantly inhibited autophagy and restored tight junction proteins. Using CHX assay, co-ip, co-localization analysis, HDOCK protein-protein docking, and Immunoelectron microscopy, we extensively proved ZO-1 and Occludin closely interact with Cav-1 and deteriorated through the autophagy pathway. Contrary to our findings, other previous studies reported that Cav-1 mediated tight junction protein Claudin-5 endocytosis and then degradation via autophagy, notably prevented the accumulation of damaged Claudin-5 in the cytoplasm and benefitted cerebral ischemia recovery 37, and in intestinal epithelial barrier inflammatory model, autophagy is necessary to maintain barrier function via inhibiting the loss of tight junction protein Occludin 52. Different tight junction proteins and their functions, and different experimental models can explain the possible reasons for these opposite conclusions. In addition, the tight junctions should be right-functioned in the cytoskeleton, their accumulation in the cytoplasm can be harmful to cell survival, and that's why autophagy-lysosome is essential to clear them out. In our study, we employed subcellular protein isolation assay and proved ZO-1 and Occludin detached from the cytoskeleton to membrane and cytoplasm after OGD/R treatment, however, the sEVs treatment reversed these trends. The sEVs treatment not only restored the ZO-1/Occludin expression level but also maintained their right subcellular location. Regarding the in-depth mechanism of how sEVs treatment can regulate the autophagy pathway and the degradation of tight junction proteins, we supposed non-coding RNAs especially microRNAs (miRNA) or circularRNAs (circRNA) are vital for their discovered therapeutic effects. Non-coding RNAs are essential for transcriptional and post-transcriptional gene expression regulation 58, consequently regulating various biological processes and diseases. In cerebral ischemia, sEVs-derived non-coding RNAs have been broadly documented to mediate acute ischemic stroke protection and long-term function recovery 59, 60. Particularly, sEVs-derived non-coding RNAs can regulate autophagy and intervene in the progression of central neural system injury diseases, including ischemic stroke 61. Kuang et, al. reported that adipose-derived mesenchymal stem cells can transfer miR-25-3p to inhibit neuron autophagy to activate neuron production in OGD-induced primary neurons and MCAo-induced mice model 41. Additionally, miR-133a-3p from BMSCs can reduce rat cerebral ischemia injury by intervening in neuron apoptosis and autophagy 62. Similarly, neuron-derived miR-21-5p enriched exosomes can promote neuroplasticity in traumatic brain injury mice model 63. Based on the current knowledge of sEVs treatment on autophagy after acute cerebral ischemia, we supposed that the autophagy inhibition on brain endothelial cells after stroke by sEVs is mediated by dozens of sEVs derived non-coding RNAs. Additional studies should be conducted to define the non-coding RNA or proteomic in BMSC-sEVs and bridge the better understanding of BMSC-sEVs therapeutic on BBB and tight junction proteins. Moreover, it should be highlighted that we proved autophagy is responsible for BBB disruption in our experimental conditions, and further studies are required to clarify the role of autophagy in different pathological stages of ischemic stroke, and autophagy outcomes in different cerebral cells subjected to ischemia. Different stroke models also matter on the pathologic mechanism and therapeutic evaluation 64, further studies should test BMSC-sEVs therapeutic and the autophagy regulation pathways in more different stroke models such as permeant MCAo models or photothrombotic models. Besides, before sEVs treatment studies are widely applied to clinics, long-term safety information should be acquired, although we tested sEVs safety in 14 days by organs H&E staining during our study, a longer period of safety verification should be performed. Also, clinical samples from stroke patients should be tested in the study for the mechanism confirmation, which further benefits the translation of sEVs application in clinical practice.

In summary, our work firstly provides a novel BMSC-sEVs therapeutic on alleviating BBB disruption post-ischemic stroke and defines the mechanism is related to Cav-1 mediated autophagic degradation of tight junction protein ZO-1 and Occludin, contributing to the long-term neurological function amelioration.

## Supplementary Material

Supplementary methods and figures.
