# Supplementary material for: BMSCs-derived small extracellular vesicles antagonize cerebral endothelial Caveolin-1 driven autophagic degradation of tight-junction proteins to protect blood-brain barrier post-stroke
Source: Int J Biol Sci. 2025 Jan 1;21(2):842–59. doi: 10.7150/ijbs.101937 (PMC11705626; doi:10.7150/ijbs.101937)
Supplement: Supplementary file 1 — Supplementary methods and figures. [file ijbsv21p0842s1.pdf]

Supplemental Material and Data of

BMSCs-derived small extracellular vesicles antagonize cerebral endothelial Caveolin-1 driven autophagic degradation of tight-junction proteins to protect blood-brain barrier post-stroke

Supplemental Methods

1. Permeability coefficient efficient of transwell assay

The permeability of b. End3 monolayer leakage was determined by the following equation:

$P_{dextran} = (RFU_{lower\ chamber} / RFU_{upper\ chamber})\ (1/ S)(V)\ (1/ t)$.

“RFU” : the fluorescent intensity of the chambers; “S” : BEC monolayer surface area; “V” : the volume of lower chamber; “t” : the time that TRITC-dextran allowed to leak.

2. siRNA and pcDNA3.1 used in this study:

Cav-1 siRNA (sense 5’-3’): CUGCGAUCCACUCUUUGAATT.

Cav-1 siRNA (antisense 5’-3’): UUCAAAGAGUGGAUCGCAGTT.

Negative ctrl siRNA (sense 5’-3’): UUCUCCGAACGUGUCACGUTT.

Negative ctrl siRNA (antisense 5’-3’): ACGUGACACGUUCGGAGAATT.

For siRNA ICV injection, 10 μg siRNA (in 1μl) was mixed with 1.1 μl lipofectamine 3000 reagent and 0.9 μl DEPC dd H_2O. The mixed cocktail was diluted to 10ul by PBS and injected to mice.

pcDNA3.1 plasmid was provided by GenePhrama Co. Ltd, Shanghai, China

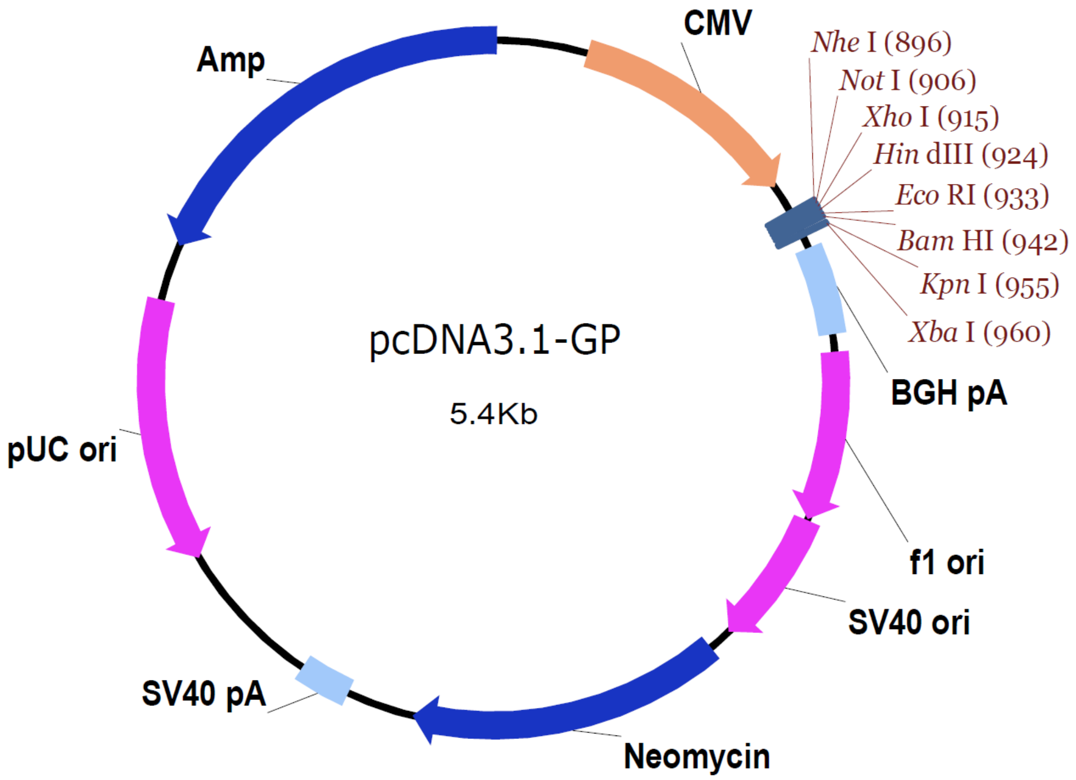

Cav-1 sequence:

atgtctgggggcaaatacgtagactccgagggacatctctacactgttcccatccgggaacagggcaacatctacaagccc

aacaacaaggccatggcagacgaggtgactgagaagcaagtgtatgacgcgcacaccaaggagattgacctggtcaac

cgcgaccccaagcatctcaacgacgacgtggtcaagattgactttgaagatgtgattgcagaaccagaagggacacacag

tttcgacggcatctggaaggccagcttcaccaccttcactgtgacaaaatattggttttaccgcttgttgtctacgatcttcggc

atcccaatggcactcatctggggcatttactttgccattctctccttcctgcacatctgggcggttgtaccgtgcatcaagagct

tcctgattgagattcagtgcatcagccgcgtctactccatctacgtccataccttctgcgatccactctttgaagctattggcaa

gatattcagcaacatccgcatcagcacgcagaaagagatatga

3. Brain infarct volume and swelling calculation

the infarct ratio was calculated by using the following equation: I=(M-S)/T. M is the area of infarct, S is the brain swelling area, and S=M-T, where M is the area of the ischemic hemisphere slices and T is the area of the non-ischemic hemisphere slices.

4. Neurological function scoring

mNSS score scale: 18 points score scale refer to the previous work by J Chen et al (PMID: 11283404):

1) Motor tests (normal: 0; maximum: 3):

Tail raising

1: Flexion of forelimb

1: Flexion of hindlimb

1: Head position alteration >10° to vertical axis in 30 s

2) Walking tests (normal: 0; maximum: 3)

0: Normal walk

1: Unable to straight walk

2: Side circling

3: Side fall down

3) Sensory tests (normal: 0; maximum: 2)

1: Placing test (visual and tactile)

2: Proprioceptive test (deep sensation, pushing the paw against the table edge to stimulate limb muscles)

4) Beam balance tests (normal: 0; maximum: 6)

0: Balances with steady posture

1: Grasps side of beam

2: Hugs the beam and one limb falls down from the beam

3: Hugs the beam and two limbs fall down from the beam, or spins on beam (>60 s)

4: Attempts to balance on the beam but falls off (>40 s)

5: Attempts to balance on the beam but falls off (>20 s)

6: Falls off: No attempt to balance or hang on to the beam (<20 s)

5) Reflexes absent and abnormal movements (normal: 0; maximum: 4)

1: Pinna reflex (head shake when touching the auditory meatus)

1: Corneal reflex (eye blink when lightly touching the cornea with cotton)

1: Startle reflex (motor response to a brief noise from snapping a clipboard paper)

1: Seizures, myoclonus

Maximum points: 18

5. Antibodies used in the study

ZO-1 (1:1000 in WB, 1:200 in IF, 61-7300, Invitrogen); Occludin (1:500 in WB, 1:150 in IF, 71-1500, Invitrogen); β-actin (1:5000, ab8227,Abcam); β-tubulin (10094-1-AP, Proteintech); Caveolin-1 (1:1000 in WB, 1:250 in IF, ab2910, Abcam); TSG 101 (1:1000, ab125011, Abcam); CD9 (1:1000, ab92726, Abcam); CD63 (1:500, ab216130, Abcam); ALIX (1:1000, ab117600, Abcam); Calpain I (1:1000, ab108400, Abcam); Calnexin (1: 1000, ab22595, Abcam); Vimentin (1:1000, ab92547, Abcam);

LC3B (1:1000 in WB, 1:250 in IF, 14600-1-AP, Proteintech); p62 (18420-1-AP, Proteintech); LAMP-1 (1:250 in IF, 67300-1-Ig, Proteintech); Tuj-1 (β-III-Tubulin(1:500 in IF, ab18207, Abcam); Lectin (Lycopersicon Esculentum (Tomato) Lectin (LEL, TL), DyLight® 488, 1:100 in IF. DL-1174-1, Vector Laboratories, Inc.) Goat Anti-Rabbit IgG H&L (HRP) (1:3000, ab6721, Abcam); Goat Anti-Mouse IgG H&L (HRP) (1:3000, ab67879, Abcam); Normal rabbit IgG (1 μg/ml, 2729S, Cell Signaling Technology); Goat Anti-mouse IgG H&L (Alexa Fluor® 488) (1:500, ab150113, Abcam); Goat Anti-Rabbit IgG H&L (Alexa Fluor® 594) (1:500, ab150080, Abcam); Goat Anti-Rabbit IgG H&L (Alexa Fluor® 488) (1:500, ab150077, Abcam).

Supplemental Figures

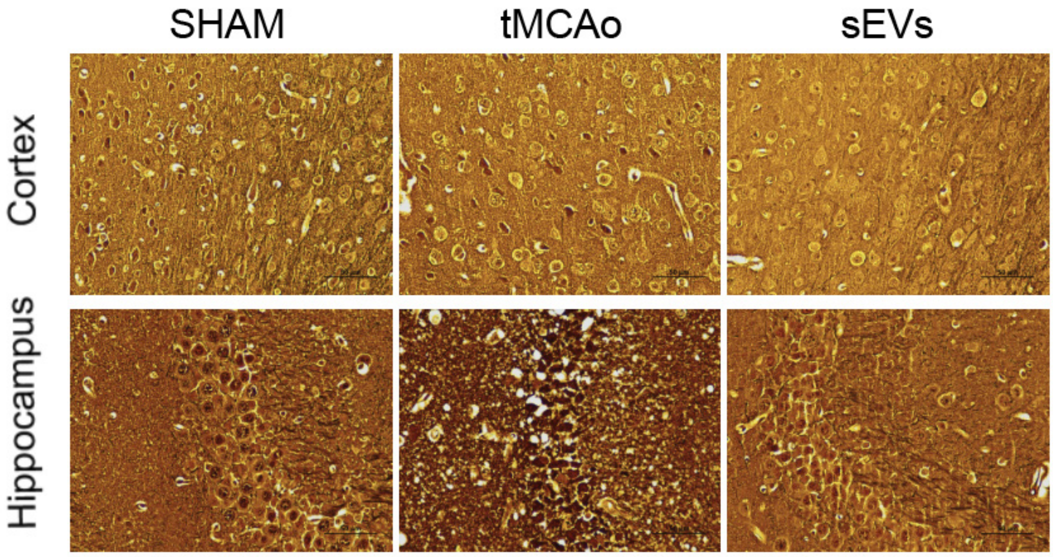

Supplemental Fig. 1 Silver glycine staining of mice cortex and hippocampus of tMCAo mice and sEVs administered mice. Scale bar: 50 µm.

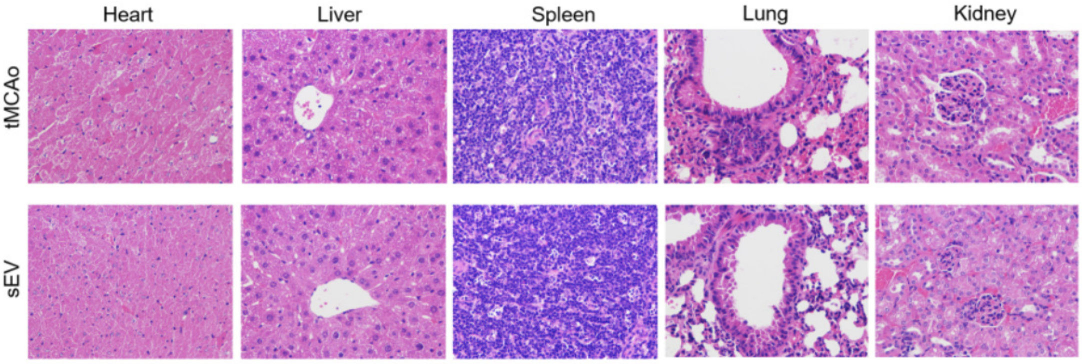

Supplemental Fig. 2 H＆E staining of mice organs of tMCAo and sEVs treating mice. Scale bar: 50 µm.

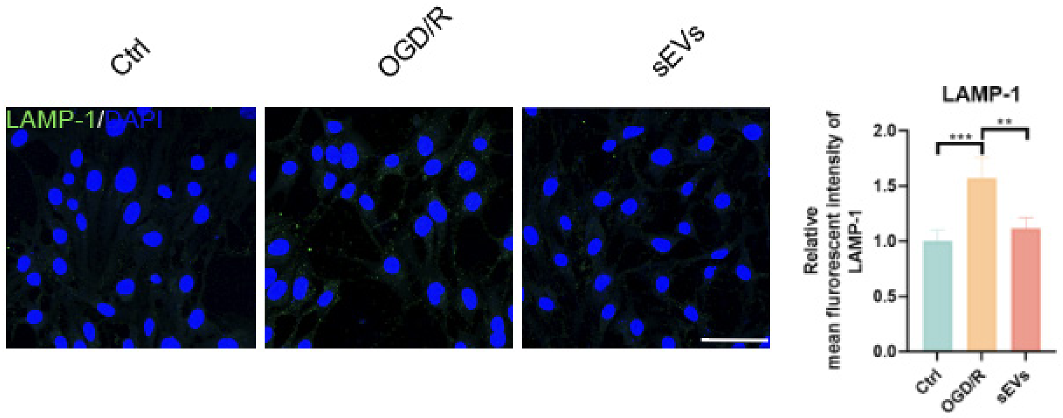

Supplemental Fig. 3 Immunofluorescence and quantification of LAMP in b. End3 cells after OGD/R and sEVs treatment (n=4). Scale bar: 50 μm. $^{***}P < 0.001$, $^{**}P < 0.01$

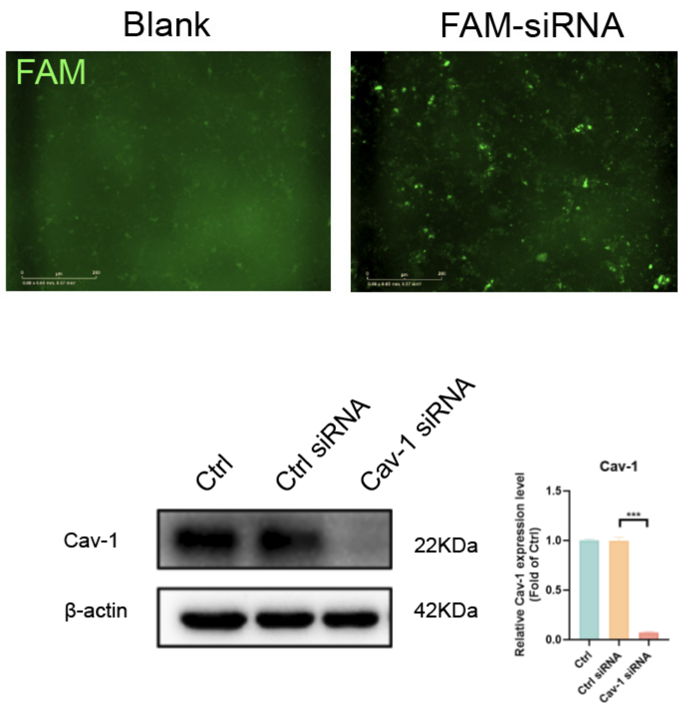

Supplemental Fig. 4 FAM- labeled siRNA transfection and verification of Cav-1 siRNA transfecion in b. End3 cells after 48h. Scale bar: 200 μm (n=3). $^{***}P < 0.001$.

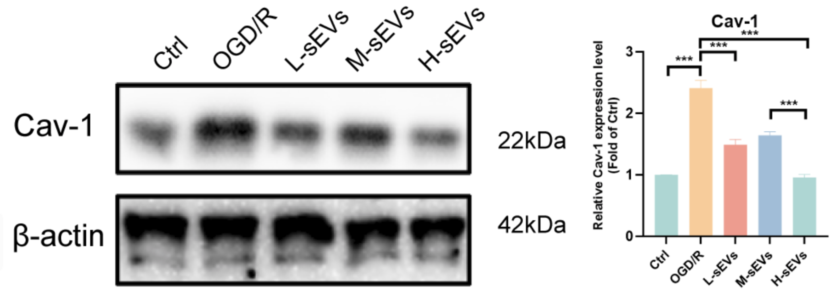

Supplemental Fig. 5 Cav-1 expression in b.End3 cells after OGD/R and different dosage sEVs treatments (n=3). $^{***}P < 0.001$.

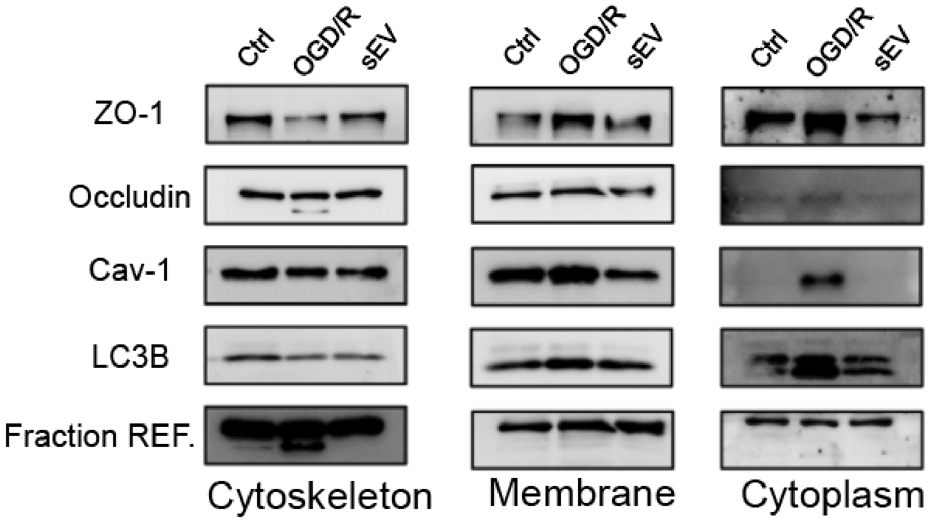

Supplemental Fig. 6 Subcellular protein expression of ZO-1, Occludin, Cav-1 and LC3B in brain endothelial cells after OGD/R and sEVs treatment.

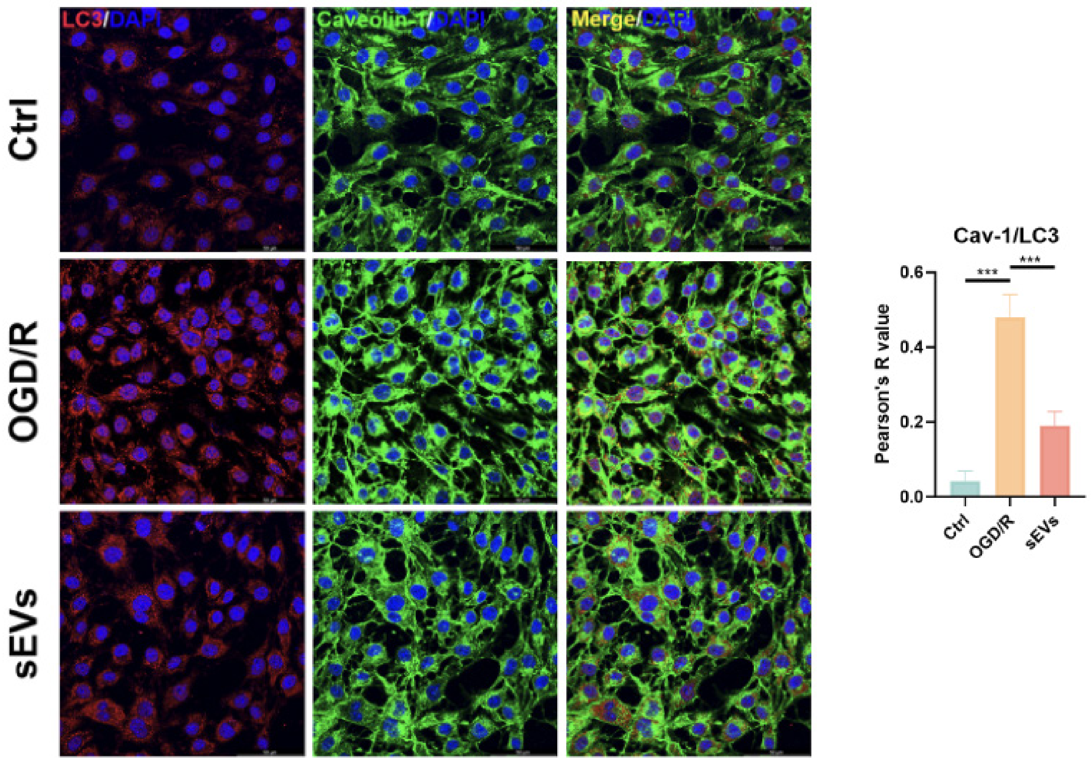

Supplemental Fig. 7 Immunofluorescence and co-localization analysis of Cav-1 and LC3B in b.End3 cells after OGD/R and sEVs treatment (n=3), Scale bar: 50 μm. $^{***}P < 0.001$.

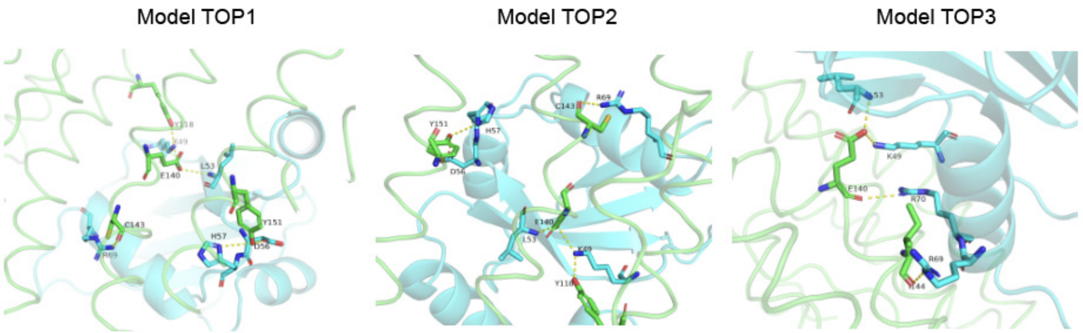

Summary of the Top 10 Models

Rank	1	2	3	4	5	6	7	8	9	10
Docking Score	-283.32	-272.63	-269.25	-269.01	-268.56	-266.72	-265.30	-264.20	-263.29	-260.98
Confidence Score	0.9350	0.9208	0.9157	0.9153	0.9146	0.9117	0.9094	0.9075	0.9060	0.9020
Ligand rmsd (Å)	374.67	346.65	308.96	313.69	316.03	374.66	350.93	335.63	375.25	349.50
Interface residues	model_1	model_2	model_3	model_4	model_5	model_6	model_7	model_8	model_9	model_10

Note: The models are ranked according to the docking scores. Please click help for the explanations of evaluation metrics

Supplemental Fig. 8 Protein-protein docking top 3 model of LC3B (Blue) and Cav-1 (Green).

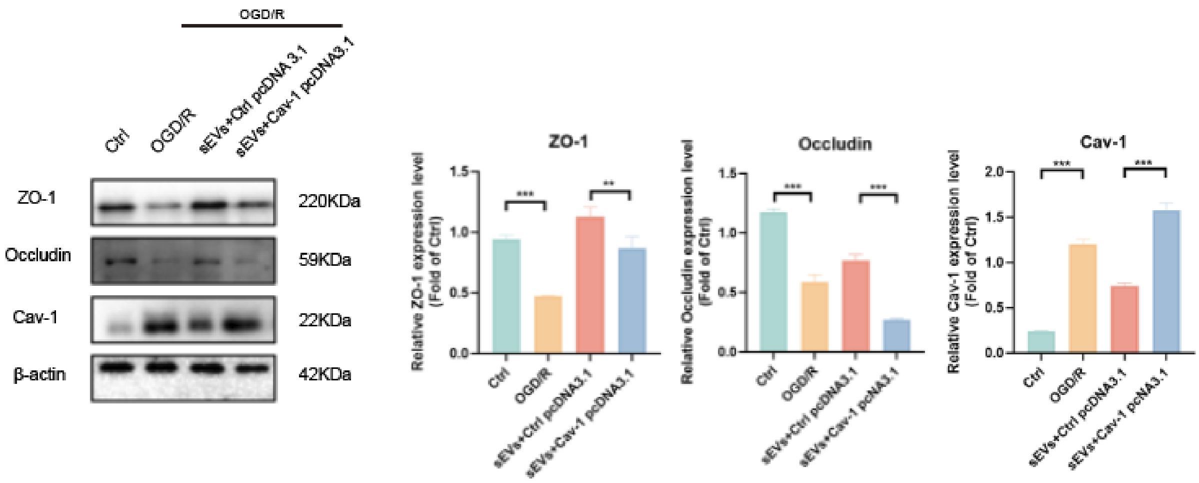

Supplemental Fig. 9 Protein expression of ZO-1, Occludin and Cav-1 after b.End3 cells subjected to OGD/R insult and sEVs treatment after Cav-1 overexpression by pcDNA3.1 (n=3). $^{***}P < 0.001$, $^{**}P < 0.01$.
